# Supplementary material for: Patterns of Intron Gain and Loss in Fungi
Source: PLoS Biol. 2004 Nov 30;2(12):e422. doi: 10.1371/journal.pbio.0020422 (PMC532390; doi:10.1371/journal.pbio.0020422)
Supplement: Table S1 — Also available at http://genes.mit.edu/NielsenEtAl/. (4.3 MB ZIP). [file pbio.0020422.st001.zip › NielsenEtAl/html/633.html]

AN8796.1.NCU01099.1.MG04881.1.FG05865.1


```
 CLUSTAL W (1.82) Multiple Sequence Alignments - Introns Inserted


Sequence 1: NCU01099.1	409 aa
Sequence 2: FG05865.1	357 aa
Sequence 3: MG04881.1	377 aa
Sequence 4: AN8796.1	332 aa
Alignment Length: 420 aa
Number Identitical Residues: 158 aa
Alignment Score (without introns) 7394


MG04881.1 	MSAGAGAGAATVPGGLGARAPDTKSQKLKKKKILLMGKSGSGKSSMRSIIFSNYLAKDTR
NCU01099.1	---MADYDQPTMGASTAAAAAHAQLKKVKKQKVLLMGKSGSGKSSMRSIIFSNYLARDTR
FG05865.1 	---MASYIDTSLAEAQPAPELLGEVKKPKKKKVLLMGKSGSGKSSMRSIIFSNYIARDTR
AN8796.1  	----------------------MDGRK-KKRKVLLMGKSGSGKSSMRSIIFSNYVAKDVR
          	                       . :* **:*:*********************:*:*.*

MG04881.1 	RL2GATIDIDLSHVKFLGNLQLNLWDCGG~QEAFMENYLSQQRQHVFSNVGVMIYVFDIE
NCU01099.1	RL~GATIDIDLSHVKFLGNLTLNLWDCGG~QEAFMENYLSQQRAHVFSNVGVLIYVFDIE
FG05865.1 	RL2GATIDIDLSHVKFLGNLTLNLWDCGG~QEAFMENYLSQQRVHVFSNVGVLIYVFDIE
AN8796.1  	RL~GATIDVEHSHVKFMGNLTLNLWDCGG2QDAFMETYLASQRGNIFSDVAVLIYVFDIE
          	** *****:: *****:*** ******** *:****.**:.** ::**:*.*:*******

MG04881.1 	SRDVDRDMATYVTILSALMQYSSGAKIFILIHKMDLIPNQTKEAVFVERVNLVRHRTEEF
NCU01099.1	SRDVDRDLATYVNIISALVQYSREAKVFVLIHKMDLIQPMTREDVFDRRVALVRRKTAEA
FG05865.1 	SRDVDRDLATYVSILSALLQYSPAAKIYILIHKMDLVVPTARESVYDERIRVVRQKTFEY
AN8796.1  	SREVERDLDTYMAIIAALREYSPHAYVFCLVHKLDLIQAEHRQRIYEERSALIRSRTEHF
          	**:*:**: **: *::** :**  * :: *:**:**:    :: :: .*  ::* :* . 

MG04881.1 	----------MHGG--------------------------------PGGQADLTPFATSI
NCU01099.1	VAIVRKQKPEVSGVGVGGLPGPGGGGGGGGAMMAGSLPSPELSTPIPDLEVEMQLFATSI
FG05865.1 	------------GNSVG----------------------------IAASSIELTPFATSI
AN8796.1  	---------------------------------------------------TIDTFGSSI
          	                                                    :  *.:**

MG04881.1 	WDQSLYKAWSEIIHDLIPSLSLIEEHLEKLGALIEAEEIQLFERTSFLVVSSWVSEVGAL
NCU01099.1	WDQSLYKAWASIIHDLVPNLSVIETQLASLGVAIDADEILLFERTSFLVVSKWTSPEGES
FG05865.1 	WDQSLYKAWASIIHDLVPNLSVIERNLANLGLAIEAEELLLFERTSFLAVSSWTSSEGQR
AN8796.1  	WDQSLYKAWAGIVHKLIPNLSVIERFLHAFAKRIDAEEVILFERSTFLTVTSVSSEIGDL
          	*********: *:*.*:*.**:**  *  :.  *:*:*: ****::**.*:.  *  *  

MG04881.1 	NPFQDRQERLSNILKAFKSSLSKYTGTPRNSEQFRDFDLKVGTGFALFITKFTTNTYILC
NCU01099.1	NPYGDRFERMSNILKAWKHTCSKYTGTPRNAEQFSDFEYKMGANFSMFVTKFTTNTYILV
FG05865.1 	NPTEDRLERMSNIMKHFKQSISRFTGTPRNAEQFIRMEHKAGPRFSLFILKFTTNTYLMV
AN8796.1  	NPIYDRHERLSNIMKAFKHCAARNTHTTPASAGFVVMHTKT-PQFNVFLGRFTDNTYIFI
          	**  ** **:***:* :*   :: * *.  :  *  :. *  . * :*: :** ***:: 

MG04881.1 	CLPPGEARFNAAKLNVRIAARLFEALDNPTNAAKAA-AAAAAASQNALGGSSVGITAGNG
NCU01099.1	CMPPGEARFNSAKLNVAAARPWFRFLDGPMIAPTAGIGSSQMLSGLAAAGGEVA------
FG05865.1 	VLPPGEARFNAAMLNCQIAIEHFRFLDGPVIQPAN-------ATTPAAA-----------
AN8796.1  	VVPPGEAAYNCAVLNTMLAREGFSKAAGAVHGDGFP---LPAPDSPDESNSN--------
          	 :***** :*.* **   *   *    ..                   ....        

MG04881.1 	EG
NCU01099.1	--
FG05865.1 	--
AN8796.1  	--
          	
```
